# Mental health literacy among primary healthcare workers in South Africa and Zambia

**DOI:** 10.1002/brb3.2807

**Published:** 2022-11-03

**Authors:** Joonas Korhonen, Anna Axelin, Dan J. Stein, Soraya Seedat, Lonia Mwape, Ronelle Jansen, Gunter Groen, Gerhard Grobler, Astrid Jörns‐Presentati, Jouko Katajisto, Mari Lahti, MEGA Consortium/Research Team

**Affiliations:** ^1^ Health and Well‐being Turku University of Applied Science Turku Finland; ^2^ Department of Nursing Science University of Turku Turku Finland; ^3^ SAMRC Unit on Risk & Resilience in Mental Disorders, Department of Psychiatry & Neuroscience Institute University of Cape Town Cape Town South Africa; ^4^ Department of Psychiatry, Faculty of Medicine and Health Sciences Stellenbosch University Cape Town South Africa; ^5^ Levy Mwanawasa Medical University School of Nursing and Midwifery Sciences Lusaka Zambia; ^6^ School of Nursing, Faculty of Health Science University of the Free State Bloemfontein South Africa; ^7^ Department of Social Work, Faculty of Business and Social Sciences University of Applied Sciences Hamburg Germany; ^8^ Department of Psychiatry, Faculty of Health Sciences University of Pretoria Tshwane South Africa; ^9^ Department of Mathematics and Statistics University of Turku Turku Finland

**Keywords:** health literacy, knowledge, low‐income country, mental health disorder, primary care

## Abstract

**Background:**

In developing countries, mental health literacy (MHL) still needs to be improved due to the high prevalence of mental disorders. It is widely recognized that MHL can improve health outcomes for both individuals and populations. Healthcare professionals’ development in MHL is crucial to the prevention of mental disorders. The aim of this study was to assess MHL of primary healthcare (PHC) workers in South Africa (SA) and Zambia and determinants thereof. Limited evidence is available on the levels of MHL among PHC workers in the sub‐Saharan Africa region, which faces a large burden of mental disorders.

**Methods:**

The study population for this cross‐sectional survey comprised PHC workers (*n* = 250) in five provinces of SA and Zambia. MHL was measured with the Mental Health Literacy Scale (MHLS). We conducted a multivariate analysis to explore determinants of MHL.

**Results:**

Results showed moderate MHL among PHC professionals, but with a wide range from low to high MHL. Knowledge‐related items had a greater dispersion than other attributes of MHL. PHC workers with more education showed a greater ability to recognize mental health‐related disorders. Those who had experience in the use of mental health‐related assessment scales or screening tools reported a higher total MHL. The results confirmed strong internal consistency for the MHLS.

**Conclusion:**

The results highlighted varying mental health perceptions and knowledge in PHC. Implementation of specifically developed formal training programs and interventions to improve MHL in PHC workers to strengthen their competence may help bridge the treatment gap.

## Graphical Abstract

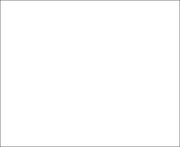

## 1 INTRODUCTION

Sub‐Saharan Africa (SSA), one of the least developed regions in the world (United Nations, 2022), faces a growing burden of mental disorders, related disabilities, and premature mortality (GBD 2017 Disease and Injury Incidence and Prevalence Collaborators, 2018; WHO, 2017). Countries in SSA must address this burden, which is costly to healthcare systems with relatively few resources (Gouda et al., 2019; Jack et al., 2014; Jörns‐Presentati et al., 2021; Munakampe, 2020; Mwape et al., 2012; Rathod et al., 2017; Stein et al., 2008), resulting in a mental health treatment gap (Ayano et al., 2017; De kock & Pillay, 2016; Sweetland et al., 2014; WHO, 2018). The treatment gap is exacerbated by socioeconomic factors such as poverty and low education (Patel, 2007; Patel & Kleinman, 2003; WHO, 2018) and, moreover, poor mental health literacy (MHL) among primary healthcare (PHC) workers in SSA (Atilola, 2016; Ayano et al., 2017). Limited investment of resources in healthcare infrastructure and the aftermath of COVID‐19 may intensify the treatment gap.

Although a great deal of work on mental health‐related knowledge has been completed regarding stigma, attitudes, and skills of PHC professionals delivering mental health services (Ayano et al., 2017; Collins et al., 2011; Davies & Lund, 2017; Kakuma et al., 2010; Petersen et al., 2019; Sweetland et al., 2014), some knowledge gaps remain to be filled (Egbe et al., 2014; Elyamani et al., 2021; Linden & Kavanagh, 2012; Saxena & Belkin, 2017; Öztaş & Aydoğan, 2021). Although PHC workers could be the key players in the promotion of public mental health literacy (MHL), as they work closely with communities (Atilola, 2016; WHO, 2018), relatively little research has been conducted in the South African and Zambian health sectors on the MHL of PHC workers. PHC workers require specific professional training on the identification and management of mental health problems (Hanlon et al., 2014; Kapungwe et al., 2011; Marais & Peterson, 2015; Mwape et al., 2010). For the promotion and effective implementation of mental health services in SSA, understanding the multiple and intersecting cultural factors that converge on mental health problems is pivotal, as these factors are linked to MHL among PHC workers and the communities in which they work and live (Atilola, 2016; Kutcher et al., 2016; 2017).

The concept of MHL originally consisted of beliefs about mental disorders that aid in their recognition, management, or prevention (Jorm et al., 1997). Early development of the concept focused on the adult population, specifically healthcare providers for raising awareness of mental health concerns (Jorm, 2012). Over time, the need to respond more broadly to the mental health problems of communities has been recognized, and the concept can also be seen as a broad set of activities that support and benefits positive mental health (Jorm 2019; Kutcher et al., 2016). MHL is closely related to health literacy, which has been recognized as a clear determinant of socioeconomic status and prosperity in life (Kutcher et al., 2016; WHO, 2013). Therefore, lack of health literacy is one factor that underlies the growing burden of (mental) health services (Sørensen et al., 2015). Although proper health literacy is a marker of economic prosperity (WHO, 2013), poor MHL among PHC professionals may contribute to the disease burden and be a barrier to adequate treatment of those in need (Ganasen et al., 2008; Loo et al., 2012; Mwape et al., 2012, Vistorte et al., 2018).

Promoting MHL among PHC professionals is a core component of successful mental healthcare integration to reach the Sustainable Development Goals for global mental health in SSA (Target 3.4; United Nations, 2020). However, MHL is a complex concept and more than just a synonym for knowledge of disorders, and it needs to be considered within specific cultural contexts (Atilola, 2016; Ganasen et al., 2008). Multicultural attitudes and beliefs affect help‐seeking behaviors in communities (Kutcher et al., 2019; Rathod et al., 2017). As SSA is a geographically and multiculturally diverse region, individuals’ beliefs vary, and these beliefs may be unique to specific cultures. These strong cultural beliefs and traditional perspectives also influence individuals’ views regarding mental health disorders (Al‐Yateem et al., 2018; Atilola, 2016; Munakampe, 2020; Mwape et al., 2012) subsequently impacting their mental health literacy.

Although the common expectation is that PHC workers should be more health literate than laypeople, previous findings showed that African PHC workers have negative attitudes toward mentally ill patients (Mosaku & Wallymahmed, 2017). Unfavorable working conditions, lack of resources, and demoralization of nurses have been reported to cultivate undesirable attitudes toward mental illness (Alburquerque‐Sendin et al, 2018). However, nurses in PHC should inform users and community members about mental health, identify mental disorders, and refer users to appropriate services (De Kock & Pillay, 2016), but unfortunately, most PHC nurses do not possess the knowledge, training, or experience to render care (Dube & Uys, 2016; Maconick et al., 2018). In addition, stigma and strong negative attitudes among PHC professionals have been found to challenge mental health support in the region (Kapungwe et al., 2011; Mosaku & Wallymahdmed, 2017). In Zambia, for instance, large numbers of PHC providers show negative stereotypes toward mentally sick people (Kapungwe et al., 2011). For that reason, an understanding of the factors that reflect perceptions of mental health issues is pivotal (Atilola, 2016).

In this study, we sought to assess the MHL of PHC workers in South Africa (SA) and Zambia including background factors, namely, demographics and educational and professional training needs. Research questions posed in the study were as follows: (1) What kind of MHL do PHC workers have in SA and Zambia? (2) What attributes of MHL are found to be the strongest and weakest among PHC workers in SA and Zambia? (3) What determinants apply to PHC workers’ MHL in SA and Zambia?

## 2 METHOD

### 2.1 Study design

We used a cross‐sectional descriptive/analytical study design (Polit & Beck, 2018) employing quantitative methods. The aim of this study was to assess the MHL of PHC workers in SA and Zambia and determinants thereof. A revised Mental Health Literacy Scale (MHLS) instrument was administered to PHC workers in SA and Zambia. The study was conducted between October 2018 and December 2019. We used the checklist of von Elm et al. (2008) on reporting cross‐sectional studies as a guide for this study. We conducted the study in association with the European Union‐funded project “MEGA‐Building Capacity by Implementing mhGAP Mobile Intervention in SADC Countries” (MEGA project; funding number 585827‐EPP‐1‐2017‐1‐FI‐EPPKA2‐CBHE‐JP) (Lahti et al., 2020).

### 2.2 Revised MHLS

Based on the original concept of MHL introduced by Jorm et al. (1997) as “a gold standard,” the MHLS of O'Connor and Casey (2015) consists of six measurable attributes of recognition, knowledge, and attitudes relating to mental health: (a) ability to recognize disorders, (b) knowing how to seek information, (c) knowledge of risk factors and causes of mental illness, (d) knowledge of self‐treatment, (e) knowledge of professional help available, and (f) attitudes that promote recognition or appropriate help‐seeking behavior (Jorm, 2000; Jorm et al., 1997; O'Connor & Casey, 2015; O'Connor et al., 2014). O'Connor and Casey (2015) psychometrically tested the original MHLS, which demonstrated excellent psychometric validity (Wei et al., 2016). We conducted this study using a revised MHLS (Korhonen et al., 2019, 2022; O'Connor & Casey, 2015) that was validated and psychometrically tested, especially for low‐ and middle‐income countries (LMICs). The revised MHLS is psychometrically sound with good construct validity and internal consistency (Korhonen et al., 2022; O'Connor & Casey, 2015; Wei et al., 2016). The revised MHLS elaborates on new information about the factors that may affect PHC workers’ provision of quality mental health care in the sub‐Saharan region.

### 2.3 The study setting and population

The study population comprised PHC workers (*n* = 306), recruited to participate in the study from five MEGA project regions and 45 clinics in SA (specifically in the Free State, Gauteng, and Western Cape Provinces) and Zambia (Lusaka and Central Provinces). We included healthcare workers in Zambia from rural, peri‐urban, and urban healthcare facilities. In line with the protocol of the MEGA project (Lahti et al., 2020), we targeted PHC workers on the front line who were the first point of contact in screening children and adolescents with mental health problems. We applied the following inclusion criteria: (a) registered or enrolled nurses or clinical officers working in PHC in the three provinces of SA and two provinces in Zambia, who (b) could speak and read English. The study excluded PHC practitioners or clinical officers who were retiring during the project (2017−2021). We performed power analysis to determine a sufficient sample size for the study population.

### 2.4 Data collection

MEGA researchers from the five local project partner universities in SA and Zambia were trained in data collection during the MEGA project partner meetings. These researchers then recruited participants from identified healthcare clinics between October 2018 and December 2019. A convenient sampling strategy was adopted with the aim of collecting data from these identified clinics situated in the Free State, Gauteng, and Western Cape Province (South Africa) as well as Lusaka and Central Provinces (Zambia). Participation in the study was voluntary and participants were required to sign a consent form after verbal and written information prior to receiving the MHLS questionnaire participation (Polit & Beck, 2018; World Medical Association, 2013). All data collected from participants were anonymous.

We conducted the study during clinics’ operating hours, and participants completed a questionnaire collecting background information as well as the MHLS consisting of 35 items. The background questionnaire included a basic survey for the demographic characteristics of the study population, and additional questions as determinants of MHL. These questions were as follows: D1, Country; D2, Working province; D3, Age group; D4, Gender; D5, Education; D6, CPD/training activity or course of mental health issues; D7, CPD/training activity or course of child and adolescent mental health issues; D8, Mental health‐related assessment scales or screening tools used in work; D9, Number of adolescents with suicidal thoughts or attempts seen per month; D10, Number of adolescents experienced trauma (e.g., rape, sexual abuse, car accidents, fire, physical abuse, etc.) seen per month; D11, Experienced problems providing mental health services in the district; and D12, Work experience. Finland, as the project coordinator, managed the data collection process, data analysis, and storage of data (paper and electronic).

### 2.5 Data analysis

We analyzed data using SPSS version 26 software. We analyzed the sample's characteristics using descriptive statistics (frequencies, percentages, medians, mean values, and standard deviation [*SD*]). To associate results between various attributes of the MHLS, we calculated the coefficient of variance (CV) to determine the level of dispersion around the means.

We formed sum variables based on the theoretical structure of the MHLS. We checked the sum variables’ reliability by calculating Cronbach's alpha coefficients and examining through item analysis the compatibility of single questions within the scale. We obtained completed cases from 250 participants, whom we included for further analysis to explore demographical and work‐related determinants of PHC workers’ MHL in SA and Zambia. Because three knowledge‐related attributes fell below the cutoff criteria (*α* ≥ .70) of internal consistency, we omitted them from the multifactor analysis of variance.

We used multifactor analysis of variance to examine the potenti effects of 12 determinants of MHL, such as demographics and training needs. The sum variables consisted of 35 items in the MHLS (main‐effect model). We used Sidak adjustments on multiple comparisons for pair‐wise comparisons. We considered statistical findings significant if the *p*‐value was ≤.05. In relation to handling missing data, we only used complete cases to compute Cronbach's alpha values and the total score for the MHLS.

## 3 RESULTS

### 3.1 Demographic characteristics of participants included in the final study

The MEGA project researchers proposed a convenience sample of PHC workers (*N* = 505), of which 306 agreed to participate. We explored Cronbach's alphas for internal consistency of the revised MHLS with the total number of recruited participants (*n* = 306). Of the scale's six attributes, three showed preferred alpha values (≥.70). The results also showed strong internal consistency (*α* = .80) for the scale's 35 items. Three knowledge‐related attributes fell below the cutoff criteria of internal consistency.

We were able to calculate the total MHLS score with complete data for 250 participants. The majority of these PHC workers were South African (65%, *n* = 162) and women (77%, *n* = 191). Of the reported cases, 59% (*n* = 143, *N* = 243) considered a diploma to be a sufficient indicator of professional education. Continuous professional development, training activities, or mental health courses had been completed by 27% of PHCs (*n* = 67, *N* = 244). Most PHC workers were young adults; 33% (*n* = 82) were 30 years old or younger. Approximately half the participants (49%, *n* = 119, *N* = 244) reported less than 10 years of work experience. The vast majority of participants (70%, *n* = 170, *N* = 243) reported experiencing problems providing mental health services in their district, and roughly half (51%, *n* = 128, *N* = 249) had used a mental health‐related assessment scale or screening tool in their work. Table 1 shows the demography of the participants.

**TABLE 1:** Demography of participants

Country	*n*	%
South Africa	162	65
Zambia	88	35
Total	250	100
Sex		
Women	191	77
Men	58	23
Total	249	100
Missing	1	
Age by group		
≤30	82	33
31−40	51	21
41−50	67	27
≥51	46	19
Total	246	100
Missing	4	
Level of professional education		
Certificate	40	17
Diploma	143	59
Degree (BA, MA, PhD)	55	23
Other	5	2
Total	243	100
Missing	7	
CPD/Training activity on mental health		
Yes	67	27
No	182	73
Total	249	100
Missing	1	

### 3.2 Results for mental health literacy of PHC workers

We explored the distribution of individual items, sum of scores, and six attributes of the revised MHLS for MHL among PHC workers (*n* = 250). The highest total score of the 35 items in the revised MHLS was 150 points (maximum score = 160). The scores ranged from 76 to 150 (mean = 122.3, *SD* = 12.4, CV = 10%) among all PHC workers. Our examination of CVs revealed that knowledge‐related questions had a greater relative dispersion around the mean. Knowing how to seek information (Q16–Q19), Knowledge of risk factors and causes of mental illness (Q9, Q10), and Knowledge of self‐treatment (Q11–Q12) had a CV of 23%, being relatively larger in comparison to the total score and other attributes of MHL. Table 2 shows the distribution of the MHLS results by attribute.

**TABLE 2:** The distribution of MHLS results among PHCs by attribute (*n* = 250)

Attribute	Minimum	Maximum	Mean	*SD*	CV
The ability to recognize disorders (Q1–Q8)	8	32	27.03	4.03	15%
Knowledge of how to seek information (Q16–Q19)	4	20	14.99	3.45	23%
Knowledge of risk factors and causes of mental illness (Q9, Q10)*	2	8	5.32	1.23	23%
Knowledge of self‐treatment (Q11–Q12)*	2	8	5.20	1.18	23%
Knowledge of professional help available (Q13–Q15)*	3	12	9.20	1.64	18%
Attitudes that promote recognition or appropriate help‐seeking behavior (stigma) (Q20–Q35)	37	79	60.58	8.19	14%
MHLS total score (Q1–Q35): minimum = 35, maximum = 160	76	150	122.32	12.42	10%
Valid *N*	*n* = 250				

* *α* < .70

We examined the distribution of results in more detail in individual responses in two groups, which employed 4‐ and 5‐point scales. In the group of 1‐ to 4‐point items measuring recognition and knowledge, the mean scores ranged from 2.13 to 3.57. A total of 12 of 15 items, including all questions regarding “Ability to recognize disorders” (Q1–Q8), had mean scores above 3.0 (minimum = 1, maximum = 4). We found the lowest mean scores, receiving less than 3 of 4, in three knowledge‐related questions (Q10, Q12, and Q15) with reversed scoring. We also found the greatest *SD* and CV of a single 4‐point question in the question related to the theme of knowledge as “a condition that would allow a mental health professional to break confidentiality” (Q15; *SD* = 1.09, CV = 44%). Table 3 shows the mean scores, *SD*, and CV of items of the 4‐point scale in the revised MHLS.

**TABLE 3:** The distribution of MHLS results by items (4‐point scale) (*n* = 250)

Item	Mean (minimum = 1, maximum = 4)	*SD*	CV
Q8: To what extent do you think it is likely that the diagnosis of “Substance Abuse Disorder” can include physical and psychological tolerance of the drug (i.e., require more of the drug to get the same effect)?	3.57	0.70	20%
Q7: To what extent do you think it is likely that the diagnosis of “Bipolar Disorder” includes experiencing periods of extremely elevated (i.e., high) and periods of depressed (i.e., low) mood?	3.56	0.80	22%
Q5: To what extent do you think it is likely that “Persistent Depressive Disorder (Dysthymia)” is a mental disorder?	3.49	0.71	20%
Q3: If someone experienced a low mood for two or more weeks, had a loss of pleasure or interest in their normal activities, and experienced changes in their appetite and sleep, then to what extent do you think it is likely they have “Major Depressive Disorder?”	3.46	0.79	23%
Q14: Mental health professionals are bound by confidentiality; however, there are certain conditions under which this does not apply. To what extent do you think it is likely that the following is a condition that would allow a mental health professional “to break confidentiality”: if a patient is at immediate risk of harm to oneself or others?	3.40	0.88	26%
Q13: To what extent do you think it is likely that “Cognitive Behavior Therapy (CBT)” is a therapy based on challenging negative thoughts and increasing helpful behaviors?	3.33	0.74	22%
Q4: To what extent do you think it is likely that “Personality Disorders” are a category of mental illness?	3.33	0.81	24%
Q2: If someone experienced excessive worry about a number of events or activities where this level of concern was not warranted, had difficulty controlling this worry, and had physical symptoms such as having tense muscles and feeling fatigued, then to what extent do you think it is likely they have “Generalised Anxiety Disorder?”	3.26	0.77	24%
Q1: If someone became extremely nervous or anxious in one or more situations with other people (e.g., in social gatherings) or performance situations (e.g., presenting at a meeting) in which they were afraid of being evaluated by others and that they would act in a way that was humiliating or feel embarrassed, then to what extent do you think it is likely they have “Social Phobia?”	3.21	0.81	25%
Q6: To what extent do you think it is likely that the diagnosis of “Agoraphobia” includes anxiety about situations (e.g., open market place) where escape may be difficult or embarrassing?	3.14	0.81	26%
Q11: To what extent do you think it would be helpful for someone to “improve their quality of sleep” if they were having difficulties managing their emotions (e.g., becoming very anxious or depressed)?	3.07	0.99	32%
Q9: To what extent do you think it is likely that in general, “women are *more* likely to experience some mental illnesses compared to men?”	3.01	0.93	31%
Q15: Mental health professionals are bound by confidentiality; however, there are certain conditions under which this does not apply. To what extent do you think it is likely that the following is a condition that would allow a mental health professional to “break confidentiality”: if patient's problem is not life‐threatening and professionals want to assist others to better support a patient (Reversed scoring)?	2.47	1.09	44%
Q10: To what extent do you think it is likely that, in general, “men are *more* likely to experience an anxiety disorder compared to women” (Reversed scoring)?	2.30	0.90	39%
Q12: To what extent do you think it would be helpful for someone to “avoid all activities or situations that made them feel anxious” if they were having difficulties managing their emotions (Reversed scoring)?	2.13	0.95	44%
Valid *N*	*N* = 250		

The mean scores of 5‐point items measuring knowledge and attitude ranged from 2.58 to 4.5. Most of the 20 items consisted of questions related to “Attitudes that promote recognition or appropriate help‐seeking behavior (stigma)” (Q20–Q35), with eight of them receiving scores ≥4.0. We found that two items that measured attitudes (Q33, Q34) had scores below 3.0. These questions were about willingness to have someone with a mental illness marry into the respondent's family (Q33; *SD* = 1.18, CV = 40%) and willingness to vote for a politician if they had suffered a mental illness (Q34; *SD* = 1.3, CV = 51%). We found that sixteen 5‐point items had an *SD* ≥ 1.000 and eight 5‐point items had a CV ≥ 30%. All items had a generally large level of dispersion around the means. Table 4 shows the mean scores, *SD*, and CV of items of the 5‐point scale.

**TABLE 4:** The distribution of MHLS results by item (5‐point scale) (*n* = 250)

Item	Mean (minimum = 1, maximum = 5)	*SD*	CV
Q24: It is best to avoid people with a mental illness so that you do not catch their illness (Reversed scoring).	4.52	0.90	20%
Q28: I believe treatment for a mental illness, provided by a mental health professional, would not be effective (Reversed scoring).	4.45	0.86	19%
Q26: Seeing a mental health professional means you are not strong enough to manage your own difficulties (Reversed scoring).	4.34	1.01	23%
Q21: A mental illness is a sign of personal weakness (Reversed scoring).	4.33	0.95	22%
Q27: If I had a mental illness, I would not seek help from a mental health professional (Reversed scoring).	4.31	1.12	26%
Q22: A mental illness is not a real medical illness (Reversed scoring).	4.19	1.07	26%
Q25: If I had a mental illness, I would tell no one (Reversed scoring).	4.14	1.03	25%
Q31: How willing would you be to make friends with someone with a mental illness?	4.05	0.94	23%
Q18: I am confident attending face to face appointments to seek information about mental illness (e.g., seeing the GP).	3.84	1.02	27%
Q16: I am confident that I know where to seek information about mental illness.	3.82	1.03	27%
Q32: How willing would you be to have someone with a mental illness start working closely with you on a job?	3.71	1.09	29%
Q17: I am confident using the computer or telephone to seek information about mental illness.	3.69	1.17	32%
Q30: How willing would you be to spend an evening socializing with someone with a mental illness?	3.69	1.09	29%
Q19: I am confident I have access to resources (e.g., GP, internet, friends) that I can use to seek information about mental illness.	3.64	1.17	32%
Q29: How willing would you be to move next door to someone with a mental illness?	3.52	1.07	30%
Q23: People with a mental illness are dangerous (Reversed scoring).	3.29	1.06	32%
Q35: How willing would you be to employ someone if you knew they had a mental illness?	3.25	1.17	36%
Q20: People with a mental illness could put themselves together if they wanted (Reversed scoring).	3.24	1.23	38%
Q33: How willing would you be to have someone with a mental illness marry into your family?	2.96	1.18	40%
Q34: How willing would you be to vote for a politician if you knew they had suffered a mental illness?	2.58	1.30	51%
Valid *N*	*N* = 250		

### 3.3 Results for the determinants of mental health literacy

We conducted variance of analysis on the scored responses (*n* = 200). We explored the analysis in comparison to background questions as determinants by calculating the total sum of the 35 items of the MHLS and three main groups of items based on attributes of the MHL: the ability to recognize disorders (Q1–Q8), knowledge of how to seek information (Q16–Q19), and attitudes that promote recognition or appropriate help‐seeking behavior (stigma; Q20–Q35). We omitted three attributes in relation to knowledge‐related questions from the analysis due to their low alpha levels (Table 2). This finding is consistent with our previous research findings on psychometric testing (Korhonen et al., 2022).

We found a statistically significant dependence in determinants relating to the level of education and the use of mental health‐related assessment scales or screening tools in work. Those who had more education were better able to recognize mental health‐related disorders (*F* = 2.869; *σ* = .038; partial *η* ^2^ = .046). In pair‐wise comparison, PHC workers with a degree‐level education had a higher mean than workers with certificates (*p* = .05).

The use of mental health‐related assessment scales or screening tools in work had a statistically significant effect on expressed attitudes and the whole MHLS instrument. In terms of mean scores, those who reported using scales or screening tools in their work showed more attitudes that promote recognition or appropriate help‐seeking behavior (stigma; *F* = 4.523; *σ* = .035; partial *η* ^2^ = .025). Moreover, they performed better in the MHL survey overall (*F* = 5.285; *σ* = .023; partial *η* ^2^ = .029). We found no statistical significance between determinants and a single attribute for knowledge of how to seek information. Neither continuous professional training/activity nor working area, as country or province, was found to be statistically significant. Table 5 shows the main results of the multifactor analysis of variance.

**TABLE 5:** Results of the multifactor analysis of variance (*n* = 200)

	*F*	*σ*	Partial *η* ^2^	*F*	*σ*	Partial *η* ^2^	*F*	*σ*	Partial *η* ^2^	*F*	*σ*	Partial *η* ^2^
Depend variable	Ability to recognise disorders			Knowledge of how to seek information			Attitudes that promote recognition or appropriate help‐seeking behaviour (stigma)			MHLS total score		
Corrected Model	0.888	.611	.099	1.852	.015	.187	0.868	.637	.097	1.040	.418	.115
intercept	199.963	.000	.530	111.069	.000	.386	265.464	.000	.600	443.926	.000	.715
Determinants												
D1: Country	0.289	.591	.002	0.132	.717	.001	0.102	.750	.001	0.004	.952	.000
D2: Working province	0.646	.631	.014	0.627	.644	.014	1.410	.232	.031	1.335	.259	.029
D3: Age Group	0.293	.831	.005	0.979	.404	.016	0.644	.588	.011	0.651	.583	.011
D4: Gender	0.407	.525	.002	2.186	.141	.012	0.028	.867	.000	0.008	.929	.000
D5: Education	2.869	.038	.046	0.901	.442	.015	0.302	.824	.005	1.003	.393	.017
D6: CPD/training activity or course of mental health issues	0.116	.734	.001	0.033	.857	.000	0.261	.610	.001	0.120	.730	.001
D7: CPD/training activity or course of child and adolescent mental health issues	1.323	.252	.007	2.700	.102	.015	0.009	.926	.000	0.037	.848	.000
D8: Mental health‐related assessment scales or screening tools used in work	1.706	.193	.010	1.881	.172	.011	4.523	.035	.025	5.285	.023	.029
D9: Number of adolescents with suicidal thoughts or attempts seen per month	0.285	.752	.003	2.411	.093	.027	0.443	.643	.005	0.484	.617	.005
D10: Number of adolescents experienced trauma (e.g., rape, sexual abuse, car accidents, fire, physical abuse, etc.) seen per month	0.483	.618	.005	2.149	.120	.024	1.213	.300	.014	1.228	.295	.014
D11: Experienced problems providing mental health services in the district	0.071	.931	.001	0.573	.565	.006	0.643	.527	.007	0.232	.794	.003
D12: Work experience	1.239	.267	.007	2.685	.103	.015	0.418	.519	.002	0.304	.582	.002
*R* squared (adjusted *R* squared)	.099 (–.013)			.187 (.086)			.097 (–.015)			.115 (.004)		

## 4 DISCUSSION

In this study, we explored the levels and determinants of MHL among PHC workers in SA and Zambia. Based on the authors’ knowledge, this is the first study that explored the level of MHL among PHC workers in SA and Zambia using the MHLS that was revised and validated (Korhonen et al., 2019, 2022).

In our findings, the scores among PHC workers ranged from 76 to 150, with the mean scores being less than a group of professionals with a Western educational background. For comparison, in the study conducted in Australia (O'Connor & Casey, 2015) mental health professionals had significantly higher MHL than the community sample. Furthermore, the results indicated strong internal consistency for the entire revised MHLS, which is also in line with our previous findings (Korhonen et al., 2022).

Mental health‐related disorders were more readily recognized by PHC workers with higher education. This is in line with Jorm (2000), who asserted that sufficient education and high MHL levels among healthcare workers make it possible for them to recognize mental disorders and promote help‐seeking behaviors. Moreover, Adu et al. (2021) found the level of education positively associated with higher MHL among Ghanaians, while it was agreed by Öztas and Aydoğan (2021) that healthcare professionals had higher MHL as a result of their education level. Linden and Kavanagh (2012) found that PHC nurses with less training as well as little exposure and experience in mental health had negative, prejudiced, and fearful attitudes toward and perceptions of people with mental health problems.

The results of this study showed that 59% of participants considered a diploma to be a sufficient indicator of professional education. Only 27% of the participants had completed continuous professional development, training activities, or mental health courses. This finding corroborates those of Marangu et al. (2021) and Petersen et al (2011), who found that most of their participants’ qualifications were at certificate and diploma levels, lacked formal mental health training, and did not participate in any mental health continuing professional development during their service. Despite the statistically significant association between the level of education and mental health, continuous professional training and mental health activity did not affect our MHL results significantly. This raises the question of the development and implementation of continuous professional training on MHL in the future to meet the needs of PCH workers. In‐service training programs for healthcare professionals may benefit from adding mental health awareness techniques. Nevertheless, additional training to improve recognition and understanding of mental health issues would benefit patients and staff (Oztas & Aydoğan, 2021).

The WHO (2007) and the International Council of Nurses already reported a scarcity of mental health education and training opportunities for nurses worldwide. Taking into account the quality and standardization of education, it is also key to improving the mental health gap, but also labor mobility. According to Bitta et al. (2017), in resource‐constrained economies, younger professionals are advantageous because training can be increased and sustained for a longer period of time.

We did not find work experience as a statistically significant determinant of MHL, but education played a bigger role in improved MHL. In line with Marangu et al. (2021), most of the study participants were young adults and women. Almost half of the participants (49%) in the present study reported working experience of less than 10 years. In a study by Al‐Yateem et al. (2018), younger nurses with less clinical experience were more knowledgeable about mental health treatment. In contrast, a systematic review found that factors associated with a lower recognition of mental illness across all demographics included young adults, illiteracy, and females (Elyamani et al., 2021).

Having an inadequate understanding of mental illness can influence mental health utilization, treatment compliance, and help‐seeking behaviors. This review by Tonsing (2018) also revealed an association between attitudes toward mental illness and help‐seeking behavior. Early detection and intervention of mental disorders are dependent on the individual's mental health literacy (Loo et al., 2012). Moreover, healthcare professionals working in primary health providing care to people with mental disorders and who are stigmatizing toward them are more likely to believe that people with mental illness will not comply with treatment (Vistorte et al., 2018).

We also found that those who had experience in the use of mental health‐related assessment scales or screening tools reported fewer stigmatizing attitudes and a higher overall MHL. However, we did not find statistical significance between the background and knowledge of how to seek information, and the rest of the three knowledge‐related questions that showed great relative dispersion fell below the cutoff criteria (*α* ≥.70), which may be caused by reversed scoring. In our study, more than half of the participants reported using a mental health‐related screening tool in their work. Marangu et al. (2021) revealed significantly low MHL results among PHC workers, evidenced by low diagnostic accuracy for serious mental health disorders. Thus, according to studies, it seems that the use of health‐related professional assessment scales and screening disorders promotes healthcare professionals’ MHL. However, the development of tools for mental health assessment alone is not useful, but training in their use would be important to better address the health challenges in the region.

Standardizing PHC workers’ mental health‐related competence proved to be a challenge in the future, as the results show a large variance in MHL levels for the entire MHLS and its attributes. All items had generally a great level of relative dispersion around the means in terms of CV. In comparison to the distribution of MHLS results among PHCs by attribute, knowledge‐related questions (of how to seek information) showed great variation (CV = 23%), with the total score having the smallest CV (10%). Generally, items of “Ability to recognize disorders” scored well on the 4‐point scale, but also had relatively large variance. Knowledge‐related questions were found to be more challenging for the respondents when examining individual items. Considering these findings, our results show heterogeneous MHLs among PHC workers, and again, raise a need for standardized mental health education.

Although significant progress has been made in improving mental health‐related knowledge, stigma, attitudes, and skills of PHC workers, knowledge gaps are still evident. Nurses are expected to be cognizant of the needs of people with mental illnesses without prejudice or discrimination (Shahif et al., 2019). Therefore, promoting MHL among PHC professionals is critical for successful integration of mental health care into mainstream healthcare services. This study's findings present factors related to MHL among PHC workers in SA and Zambia. Despite inadequate knowledge and competence of healthcare professionals to screen and care for people with mental health problems, as well as poor attitudes toward mental health problems, we found gaps in their MHL. By identifying these gaps, we provide an opportunity for and guide potential future research in mental health services in PHC.

## 5 CONCLUSION

Our findings reflected MHL among PHC in SA and Zambia as moderate, but it varies according to educational background. One of the prominent findings of this study was the significance of education and guidelines for screening mental health disorders that promotes MHL. Education may foster PHC workers’ ability to recognize mental health disorders, while professionals’ routine assessment of mental health can reduce stigma and strengthen MHL overall. Therefore, it is important to increase PHC workers’ mental health knowledge and strengthen their skills through training. Standardizing and enhancing their mental health competence is critical for successful integration of mental health care into PHC services and will improve the response to mental health problems in SA and Zambia.

## 6 LIMITATIONS

This study has limitations that should be considered when interpreting the results. First, although the study included a small (*n* = 250) but statistically relevant sample, it consisted of only nurses and clinical officers. Extending the participation to other healthcare disciplines may provide more insights into the levels and determinants of MHL among PHC professionals in SSA. Second, based on our findings, we suggest using the revised MHLS to measure MHL using the scale's total score, as three of the knowledge‐related attributes that mainly had reversed scoring system, and as an obvious reason, fell below the appropriate level of internal consistency, nor can individual variables be used to draw conclusions about MHL. Last, no gold standards have been introduced regarding the measurement of MHL. From the outset, we acknowledged the challenge in determining which areas of the revised MHLS performed better, which is in tandem with Wei et al. (2015), who asserted that it was difficult to determine the study results’ value and impossible to conduct cross‐study comparisons of different interventions, especially considering the high proportion of nonvalidated measures being applied. The MHLS results may vary between different groups, and no strict conclusions can be made from the presented results.

## CONFLICT OF INTEREST

The authors declare no conflict of interest.

## Data Availability

Research data are not shared.
